# Association between insulin resistance, hyperglycemia, and coronary artery disease according to the presence of diabetes

**DOI:** 10.1038/s41598-019-42700-1

**Published:** 2019-09-02

**Authors:** Young-Rak Cho, Soe Hee Ann, Ki-Bum Won, Gyung-Min Park, Yong-Giun Kim, Dong Hyun Yang, Joon-Won Kang, Tae-Hwan Lim, Hong-Kyu Kim, Jaewon Choe, Seung-Whan Lee, Young-Hak Kim, Shin-Jae Kim, Sang-Gon Lee

**Affiliations:** 1 Division of Cardiology, Dong-A University Hospital, Busan, Republic of Korea; 2 Division of Cardiology, Ulsan University Hospital, University of Ulsan College of Medicine, Ulsan, Republic of Korea; 3 Division of Radiology, Asan Medical Center, University of Ulsan College of Medicine, Seoul, Republic of Korea; 4 Division of Health Screening and Promotion Center, Asan Medical Center, University of Ulsan College of Medicine, Seoul, Republic of Korea; 5 Division of Cardiology, Asan Medical Center, University of Ulsan College of Medicine, Seoul, Republic of Korea

**Keywords:** Calcification, Diabetes complications

## Abstract

This study evaluated the relationship of insulin resistance (IR) and glycemic control status to the presence and severity of coronary artery disease (CAD) according to diabetes. The relationship of IR parameters including homeostatic model assessment of IR (HOMA-IR), triglyceride-glucose (TyG) index, and triglyceride-to-high density lipoprotein cholesterol ratio (TG/HDL), and hemoglobin A1C (HbA1C) level to CAD and obstructive CAD was evaluated in 5,764 asymptomatic subjects who underwent coronary computed tomographic angiography. Non-diabetics (n = 4768) and diabetics (n = 996) were stratified into four groups based on the quartiles of HOMA-IR and the TyG index and were grouped based on the TG/HDL cut-offs of 3.5, respectively. CAD and obstructive CAD were defined as the presence of any plaques and plaques with ≥50% stenosis, respectively. The prevalence of CAD (59.0% vs. 39.0%) and obstructive CAD (15.0% vs. 6.6%) was higher in diabetic than in non-diabetic patients (p < 0.001, respectively). In non-diabetic patients, the adjusted odds ratio for both CAD and obstructive CAD significantly increased, but only with higher TyG index quartiles. Unlike non-diabetics, the adjusted odds ratio for obstructive CAD significantly increased in diabetic patients with a TG/HDL level ≥ 3.5. The HbA1C, rather than IR parameters, was independently associated with both CAD and obstructive CAD in diabetics. In conclusion, among IR parameters, TyG index was independently associated with the presence of CAD and obstructive CAD in non-diabetic patients. In contrast, the glycemic control status, rather than IR, was importantly related to both CAD and obstructive CAD in established diabetic patients.

## Introduction

Coronary artery disease (CAD) is a leading cause of morbidity and mortality worldwide^1^. Previous studies have revealed that insulin resistance (IR) is significantly related to the development and progression of coronary atherosclerosis, adverse plaque characteristics, and an increased risk of adverse cardiovascular (CV) outcomes^2–4^. These results may be associated with an increase in the prevalence of diabetes because IR is a major characteristic of diabetes. Previous several studies reported the impact of IR and glycemic control status on CAD in symptomatic non-diabetic patients who referred to invasive coronary angiography^5,6^. However, despite recent strong evidence for the significance of strict glycemic control in established diabetic patients^7–10^, there are limited data on the relationship of IR and glycemic status with the presence and severity of CAD according to diabetic status, especially in asymptomatic general population.

The hyperinsulinemic-euglycemic clamp is the gold standard for measuring IR. However, it is not practical in clinical settings because this method is invasive, laborious, and expensive. In clinical practice, the homeostasis model assessment of insulin resistance (HOMA-IR) was developed as a more convenient way to measure IR and has been used widely. Recently, both the triglyceride-glucose (TyG) index and triglyceride (TG)-to-high density lipoprotein cholesterol (HDL) ratio (TG/HDL) have been suggested as new ways to measure IR^11,12^. Therefore, in the present study, we evaluated the relationship of the HOMA-IR, TyG index, TG/HDL, and hemoglobin A1C (HbA1C) level with the presence of CAD and obstructive CAD according to diabetic status in asymptomatic 7,129 subjects using non-invasive coronary computed tomographic angiography (CCTA).

## Methods

### Study population

A total of 9,269 self-referred, consecutive subjects aged ≥ 20 years underwent CCTA for general health examination at the Asan Medical Center between January 2007 and December 2011. Among these subjects, 7,129 took part in this study. We excluded subjects with (1) a previous history of angina or myocardial infarction (n = 336); (2) abnormal electrocardiographic findings including a pathologic Q wave, ischemic change of ST segments or T wave, and left bundle branch block (n = 205); (3) structured heat disease (n = 49); (4) insufficient medical records (n = 670); (5) history of percutaneous coronary intervention (n = 5); (6) history of open heart surgery (n = 5); (7) history of cardiac procedures including atrial septal defect device closure (n = 4), percutaneous mitral valvuloplasty (n = 2), permanent pacemaker (n = 2), patent ductus arteriosus device closure (n = 1), and patent foramen ovale (n = 1); and (8) renal insufficiency (n = 1). Finally, 5,764 subjects were enrolled. All participants were stratified into four groups based on their HOMA-IR and TyG index quartiles and into two groups according to their diabetic status, based on a TG/HDL cutoff point of 3.5, which is well-known to be highly correlated with IR^12,13^. The cutoff values for the HOMA-IR and TyG index quartiles in non-diabetic patients and diabetic patients are described in Supplementary Table 1. We extracted data on the participants’ medical histories from their responses to a systemized self-reported questionnaire. Hypertension was defined as systolic blood pressure more than 140 mmHg or diastolic blood pressure more than 90 mmHg, previous diagnosis of hypertension, or anti-hypertensive medication. Hyperlipidemia was defined as a total cholesterol level more than 240 mg/dL or anti-hyperlipidemic treatment. Diabetes was defined as a fasting glucose more than 126 mg/dL, HbA1C more than 6.5%, or antidiabetic medications^14,15^. The protocol of present study was approved by the institutional review board of Asan Medical Center. All methods were performed in accordance with the relevant guidelines and regulations.

### Clinical measurements

Weight and height were measured during the subjects wore light clothing without shoes. The body mass index was calculated as weight (kg)/height (m^2^). Blood pressure of the right arm was measured using an automatic manometer after resting for at least more than 5 minutes. After the participants fasted overnight, blood samples were collected and analyzed in the central laboratory. The measurement of total cholesterol, TG, HDL cholesterol, and low-density lipoprotein cholesterol levels was performed with an enzymatic colorimetric method, using a Toshiba 200FR Neo (Toshiba Medical System Co., Ltd., Tokyo, Japan). Fasting glucose levels were measured with an enzymatic colorimetric method using a Toshiba 200 FR auto-analyzer (Toshiba). HbA1C levels were measured with ion-exchange high-performance liquid chromatography (Bio-Rad Laboratories, Inc., Hercules, CA, USA). All measurements of enzyme activities were performed at 37 °C. The HOMA-IR was calculated using the following formula: HOMA-IR = fasting insulin (μU/mL) × fasting plasma glucose (mg/dL)/405^16^. The TyG index was calculated as ln (fasting triglycerides [mg/dL] × fasting glucose [mg/dL]/2)^17^.

### Acquisition and analysis of CCTA images

CCTA was performed using dual-source CT (Somatom Definition, Siemens, Erlangen, Germany) or single-source 64-slice CT (LightSpeed VCT, GE, Milwaukee, WI, USA). Subjects with an initial heart rate more than 65 bpm received an oral dose of 2.5 mg of bisoprolol (Concor, Merck, Darmstadt, Germany) 1 h before the CT examination, if beta blockers were not contraindicated. Prospective electrocardiography-triggering mode or the retrospective electrocardiography-gating mode with electrocardiography-based tube current modulation was used in CT scanning. Two puffs (2.5 mg) of isosorbide dinitrate (Isoket spray, Schwarz Pharma, Monheim, Germany) were used before contrast was injected. The injection of 60–80 mL of iodinated contrast (Iomeron 400, Bracco, Milan, Italy) was done at a dose of 4 mL/s, followed by a 40 mL of saline flush during CCTA. A standard scanning protocol was used, and the tube voltage and current time were adjusted according to the body size as following: 100 or 120 kVp tube voltage; 240 to 400 mAs per rotation (dual-source CT); and 400 to 800 mA (64-slice CT) tube current. All CCTA images were analyzed by specialized CV radiologists (DHY, JWK, and THL) with a workstation (Volume Wizard, Siemens; or Advantage Workstation, GE).

According to the Society of Cardiovascular Computed Tomography guidelines, a 16-segment coronary artery tree model was used. Plaque was defined as structures > 1 mm^2^ within or adjacent to the lumen. Plaque with calcified tissue involving more than 50% of the plaque area (density > 130 HU) were classified as calcified, plaque with less than 50% calcium were classified as mixed, and plaque without calcium were classified as non-calcified lesions. The contrast-enhanced portion of the coronary lumen was semi-automatically traced at the maximal stenotic site, and this value was compared to the mean value of the proximal and distal reference sites. Stenosis more than 50% was defined as obstructive. CAD was defined as the presence of any plaques, and obstructive CAD was defined as the presence of obstructive plaques.

### Statistical analysis

Continuous variables are expressed as the mean ± standard deviation. Categorical variables are presented as absolute values and proportions. One-way analysis of variance or Student’s t-test was used for continuous variables, as appropriate. The χ^2^ test or Fisher exact test was used for categorical variables, as appropriate. A univariate logistic regression analysis was performed for identifying the association between clinical variables and coronary atherosclerotic parameters. A multivariate logistic regression analysis was used to identify the independent impact of the IR parameters and HbA1C level on CAD and obstructive CAD. The forced-entry method was used to enter independent variables into the multivariate regression analysis. All statistical analyses were performed using the Statistical Package for the Social Sciences, version 19 (SPSS, Chicago, IL, USA), and a p-value < 0.05 was considered significant for all analyses.

### Ethics approval and consent to participate

The protocol of present study was approved by the institutional review board of Asan Medical Center, and informed consent was obtained from each participant.

## Results

### Baseline characteristics

**Figure 1:**
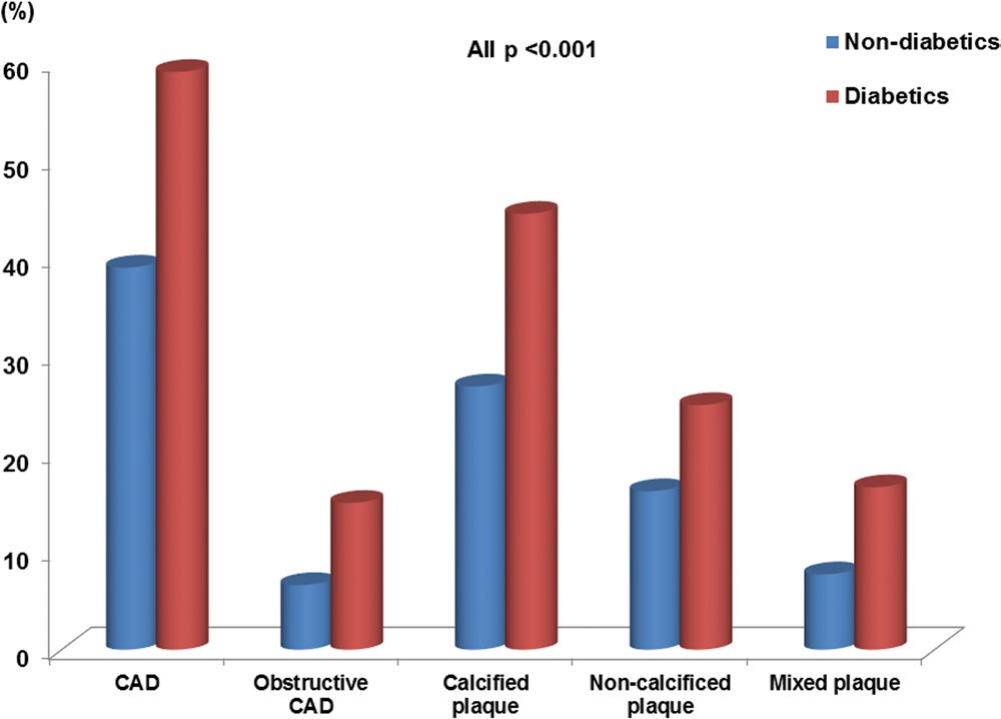
Comparison of non-diabetic patients and diabetic patients in terms of coronary atherosclerosis.

Table 1 shows the clinical characteristics of participants according to their diabetic status. The mean age was 53.7 ± 7.7 years, and 4,214 (73.1%) were male. Age; body mass index; waist circumference; systolic and diastolic blood pressure; total cholesterol, TG, fasting glucose, TG/HDL, and HbA1C levels; HOMA-IR; and TyG index were significantly higher in diabetic patients than in non-diabetic patients. The incidence of male sex, hypertension, dyslipidemia, and current smoking status was significantly higher in diabetic patients than in non-diabetic patients. The HDL and low-density lipoprotein cholesterol levels were significantly lower in diabetic patients than in non-diabetic patients. Diabetic patients had significantly higher incidences of CAD (59.0% vs. 39.0%) and obstructive CAD (15.0% vs. 6.6%) than did non-diabetic patients (all p < 0.001). Additionally, the incidences of calcified plaque (44.5% vs. 26.9%), non-calcified plaque (25.0% vs. 16.2%), and mixed plaque (16.6% vs. 7.7%) were significantly higher in diabetic patients than in non-diabetic patients (all p < 0.001) (Fig. 1).

**Table 1:** Baseline characteristics.

	Non-diabetics (n = 4768)	Diabetics (n = 996)	p
Age, yrs	53.2 ± 7.7	55.8 ± 7.6	<0.001
Male, n (%)	3393 (71.2)	821 (82.4)	<0.001
Body mass index, kg/m^2^	24.5 ± 2.9	25.4 ± 3.0	<0.001
Waist circumference, cm	85.4 ± 8.3	88.9 ± 8.0	<0.001
Systolic blood pressure, mmHg	119.0 ± 12.8	122.8 ± 13.5	<0.001
Diastolic blood pressure, mmHg	76.2 ± 10.4	77.9 ± 10.0	<0.001
Hypertension, n (%)	1580 (33.1)	523 (52.5)	<0.001
Dyslipidemia, n (%)	1373 (28.8)	438 (44.0)	<0.001
Current-smoking, n (%)	1083 (22.7)	297 (29.8)	<0.001
Total cholesterol, mg/dL	197.0 ± 33.2	187.0 ± 38.1	<0.001
TG, mg/dL	129.6 ± 77.3	159.4 ± 108.2	<0.001
HDL cholesterol, mg/dL	53.7 ± 13.5	50.2 ± 12.4	<0.001
LDL cholesterol, mg/dL	122.9 ± 29.2	113.4 ± 33.1	<0.001
Creatinine, mg/dL	0.90 ± 0.16	0.91 ± 0.16	0.223
Fasting glucose, mmol/L	5.5 ± 0.5	7.5 ± 1.9	<0.001
HOMA-IR	1.96 ± 1.23	3.38 ± 4.26	<0.001
TyG index	8.63 ± 0.53	9.09 ± 0.65	<0.001
TG/HDL	2.73 ± 2.13	3.52 ± 2.81	<0.001
HbA1C, %	5.5 ± 0.4	6.8 ± 1.2	<0.001

Values are given as the mean ± standard deviation or number (%).

*CAD* coronary artery disease, *HbA1C* hemoglobin A1C, *HDL* high-density lipoprotein, *HOMA-IR* homeostatic model assessment of insulin resistance, *LDL* low-density lipoprotein; *TG* triglyceride; *TyG* triglyceride-glucose.

### CCTA findings according to IR parameters that were based on diabetic status

Table 2 shows the incidence of coronary atherosclerotic parameters that were based on the HOMA-IR, TyG quartile, and TG/HDL cutoff value of 3.5 in non-diabetic patients and diabetic patients. In non-diabetic patients, the incidences of CAD and obstructive CAD were significantly different according to the HOMA-IR and TyG index quartile and TG/HDL cutoff value of 3.5. Furthermore, the incidences of calcified, non-calcified, and mixed plaque were significantly different among the HOMA-IR, TyG index, and TG/HDL groups. In contrast, the incidences of CAD and obstructive CAD in diabetic patients were significantly different in only the HOMA-IR quartiles. In diabetic patients, the incidence of calcified plaque (neither non-calcified nor mixed plaque) was significantly different according to the HOMA-IR quartile.

**Table 2:** CCTA findings according to IR parameters.

	HOMA-IR quartile					TyG index quartile					TG/HDL		
	I (lowest)	II	III	IV (highest)	P	I (lowest)	II	III	IV (highest)	p	<3.5	≥3.5	p
**Non-diabetics**	n = 1194	n = 1191	n = 1193	n = 1190		n = 1197	n = 1188	n = 1193	n = 1190		n = 3638	n = 1130	
CAD, n (%)	382 (32.0)	453 (38.0)	492 (41.2)	532 (44.7)	<0.001	352 (29.4)	441 (37.1)	528 (44.3)	538 (45.2)	<0.001	1339 (36.8)	520 (47.0)	<0.001
Obstructive CAD, n (%)	54 (4.5)	80 (6.7)	77 (6.5)	103 (8.7)	0.001	41 (3.4)	77 (6.5)	102 (8.5)	94 (7.9)	<0.001	221 ((6.1)	93 (8.2)	0.011
Plaque characteristics, n (%)													
Calcified plaque	266 (22.3)	306 (25.7)	343 (28.8)	366 (30.8)	<0.001	251 (21.0)	313 (26.3)	366 (30.7)	351 (29.5)	<0.001	949 (26.1)	332 (29.4)	0.029
Non-calcified plaque	147 (12.3)	197 (16.5)	207 (17.4)	221 (18.6)	<0.001	128 (10.7)	172 (14.5)	220 (18.4)	252 (21.2)	<0.001	525 (14.4)	247 (21.9)	<0.001
Mixed plaque	66 (5.5)	82 (6.9)	102 (8.5)	117 (9.8)	<0.001	53 (4.4)	83 (7.0)	105 (8.8)	126 (10.6)	<0.001	242 (6.7)	125 (11.1)	<0.001
**Diabetics**	n = 249	n = 251	n = 247	n = 249		n = 250	n = 248	n = 250	n = 248		n = 632	n = 364	
CAD, n (%)	144 (57.8)	133 (53.0)	138 (55.9)	173 (69.5)	<0.001	137 (54.8)	144 (58.1)	158 (63.2)	149 (60.1)	0.277	367 (58.1)	221 (60.7)	0.414
Obstructive CAD, n (%)	32 (12.9)	23 (9.2)	43 (17.4)	51 (20.5)	0.002	34 (13.6)	28 (11.3)	40 (16.0)	47 (19.0)	0.098	84 (13.3)	65 (17.9)	0.052
Plaque characteristics, n (%)													
Calcified plaque	107 (43.0)	101 (40.2)	96 (38.9)	139 (55.8)	<0.001	104 (41.6)	112 (45.2)	125 (50.0)	102 (41.1)	0.165	285 (45.1)	158 (43.4)	0.606
Non-calcified plaque	61 (24.5)	55 (21.9)	60 (24.3)	73 (29.3)	0.278	59 (23.6)	55 (22.2)	61 (24.4)	74 (29.8)	0.216	146 (23.1)	103 (28.3)	0.068
Mixed plaque	40 (16.1)	33 (13.1)	44 (17.8)	48 (19.3)	0.287	36 (14.4)	35 (14.1)	50 (20.0)	44 (17.7)	0.230	98 (15.5)	67 (18.4)	0.236

Values are given as the mean ± standard deviation or number (%).

*CAD* coronary artery disease, *CCTA* coronary computed tomographic angiography, *HDL* high-density lipoprotein, *HOMA-IR* homeostatic model assessment of insulin resistance, *TG* triglyceride, *TyG* triglyceride-glucose.

### Association between IR parameters and coronary atherosclerosis according to diabetic status

Table 3 shows the result of the multivariate regression analysis of the relationship of the HOMA-IR, TyG index, and TG/HDL with coronary atherosclerosis according to the presence of diabetes. In non-diabetic patients, the risk of obstructive CAD was higher in HOMA-IR group IV (highest) than it was in group I (lowest). The risk of CAD was higher in TyG index groups III and IV than in TyG index group I, and that of obstructive CAD was higher in groups II, III, and IV than it was in group I. The risk of CAD was higher in patients with a TG/HDL level ≥ 3.5 than it was in those with a TG/HDL level < 3.5. In diabetic patients, the risk of obstructive CAD was higher in those with a TG/HDL level ≥ 3.5 than it was in those with a TG/HDL level < 3.5. The risk of CAD and obstructive CAD was not significantly different among the HOMA-IR and TyG index quartile groups. The result of the univariate logistic regression analysis of the association between clinical variables and coronary plaque according to diabetic status is described in Supplementary Table 2.

**Table 3:** Association among the HOMA-IR, TyG index, TG/HDL, and coronary atherosclerosis according to diabetic status.

	Non-diabetics				Diabetics			
	CAD		Obstructive CAD		CAD		Obstructive CAD	
	OR (95% CI)	P	OR (95% CI)	p	OR (95% CI)	p	OR (95% CI)	p
By HOMA-IR quartile								
I	1		1		1		1	
II	1.081 (0.892–1.309)	0.426	1.287 (0.886–1.869)	0.185	0.815 (0.550–1.207)	0.307	0.768 (0.423–1.396)	0.387
III	1.168 (0.961–1.421)	0.119	1.242 (0.846–1.822)	0.269	0.892 (0.593–1.342)	0.584	1.512 (0.877–2.607)	0.137
IV	1.203 (0.976–1.483)	0.083	1.564 (1.057–2.313)	0.025	1.413 (0.889–2.243)	0.143	1.617 (0.907–2.882)	0.103
By TyG index quartile								
I	1		1		1		1	
II	1.112 (0.916–1.351)	0.283	1.661 (1.111–2.483)	0.013	1.300 (0.873–1.937)	0.197	0.929 (0.531–1.626)	0.797
III	1.243 (1.020–1.514)	0.031	1.938 (1.304–2.881)	0.001	1.391 (0.922–2.099)	0.116	1.329 (0.776–2.277)	0.300
IV	1.299 (1.058–1.594)	0.012	1.861 (1.232–2.811)	0.003	1.160 (0.747–1.802)	0.509	1.458 (0.830–2.564)	0.190
By cut-off 3.5 of TG/HDL								
<3.5	1		1		1		1	
≥3.5	1.176 (1.007–1.373)	0.041	1.222 (0.930–1.605)	0.149	1.034 (0.764–1.401)	0.827	1.504 (1.015–2.230)	0.042

Adjusted for age, sex, waist circumference, hypertension, dyslipidemia, current smoking status, and HbA1c level.

*CAD* coronary artery disease, *CI* confidence interval, *HbA1C*, hemoglobin A1C; *HDL* high-density lipoprotein, *HOMA-IR* homeostatic model assessment of insulin resistance, *OR* odds ratio, *TG* triglyceride, *TyG* triglyceride-glucose.

### Association between the HbA1C level and coronary atherosclerosis according to diabetic status

Table 4 shows the result of the multivariate regression analysis of the association between the HbA1C level and coronary atherosclerosis according to diabetic status. After adjusting for confounding clinical factors, we found that the HbA1C level did not have a significant association with the presence of CAD and obstructive CAD in non-diabetic patients. However, the HbA1C level was independently associated with an increased risk of CAD and obstructive CAD in diabetic patients.

**Table 4:** Association between the HbA1C level and coronary atherosclerosis according to diabetic status.

	Non-diabetics				Diabetics			
	CAD		Obstructive CAD		CAD		Obstructive CAD	
	OR (95% CI)	P	OR (95% CI)	p	OR (95% CI)	p	OR (95% CI)	p
Model 1	1.682 (1.438–1.968)	<0.001	1.817 (1.334–2.474)	<0.001	1.227 (1.095–1.375)	<0.001	1.395 (1.234–1.577)	<0.001
Model 2	1.081 (0.904–1.291)	0.394	1.227 (0.882–1.708)	0.224	1.271 (1.127–1.434)	<0.001	1.457 (1.276–1.663)	0.001
Model 3	1.040 (0.868–1.245)	0.671	1.160 (0.831–1.620)	0.383	1.226 (1.085–1.387)	0.001	1.440 (1.259–1.648)	0.001
Model 4	1.050 (0.878–1.257)	0.591	1.159 (0.831–1.615)	0.385	1.275 (1.119–1.454)	0.001	1.404 (1.218–1.619)	0.001
Model 5	1.071 (0.896–1.280)	0.454	1.197 (0.860–1.667)	0.287	1.274 (1.126–1.441)	<0.001	1.422 (1.243–1.625)	<0.001

*CAD* coronary artery disease, *CI* confidence interval, *HbA1C* hemoglobin A1C, *HDL* high-density lipoprotein, *HOMA-IR* homeostatic model assessment of insulin resistance, *OR* odds ratio, *TG* triglyceride, *TyG* triglyceride-glucose.

Model 1: Unadjusted.

Model 2: Adjusted for age, sex, waist circumference, hypertension, dyslipidemia, and current smoking.

Model 3: Adjusted for age, sex, waist circumference, hypertension, dyslipidemia, current smoking, and HOMA-IR.

Model 4: Adjusted for age, sex, waist circumference, hypertension, dyslipidemia, current smoking, and TyG index.

Model 5: Adjusted for age, sex, waist circumference, hypertension, dyslipidemia, current smoking, and TG/HDL.

## Discussion

In this study, we investigated the relationship of IR parameters and glycemic status with the presence and severity of CAD according to the presence of diabetes in asymptomatic individuals, using CCTA. To our knowledge, this is the first study to investigate such a relationship. Based on our analysis, we found that the IR, especially TyG index, was independently associated with the presence of CAD and obstructive CAD in non-diabetic patients. In contrast, the HbA1C level, which is a marker of glycemic control, is more related to CAD and obstructive CAD in diabetic patients than IR parameters are. Thus, regarding the presence and severity of CAD, we could identify the significance of IR in non-diabetics and that of glycemic control in established diabetics in asymptomatic large population.

The significance of IR in the development of CAD is well established. Using the Archimedes model of dyslipidemia in diabetic patients, Eddy *et al*. reported that IR was the single most frequent cause of CAD^18^. The San Antonio Heart Study revealed there was an independent association between IR and the risk of CAD^19^. In the Bruneck study, Bonora *et al*. reported that IR was associated with symptomatic, subsequent CAD, irrespective of the traditional CV risk factors, in the general population^20^. A number of previous studies revealed that elevated IR was associated with an increased risk of CV events in non-diabetic patients^21–24^. However, there has been conflicting evidence about the relationship between IR and the risk of CV events in established diabetic patients. The Veterans Affairs HDL Intervention Trial^22^ and Verona Diabetes Complications Study^25^ reported that the HOMA-IR was associated with an increased risk of future CV events. However, the UK Prospective Diabetes Study did not observe a significant relationship between the HOMA-IR and the risk of CV events^26^. Considering these results, it is important to identify the relationship between IR and the presence and severity of CAD in the general population according to whether diabetes is present.

Several surrogate markers of IR for predicting diabetes and CAD have been investigated in clinical practice. Traditionally, the HOMA-IR has been used to measure IR^27^. Moreover, several studies reported that a TG/HDL level ≥ 3.5 is highly correlated with IR and atherogenic dyslipidemia, and can predict diabetes^12,13^. Recently, two studies reported that the TyG index is closely correlated with the HOMA-IR^28,29^. Furthermore, other studies reported that the value of the TyG index to predict IR is better than that of the HOMA-IR^30,31^. In the present study, the incidences of CAD and obstructive CAD were significantly higher in diabetic patients than in non-diabetic patients. In non-diabetic patients, only the TyG index was associated with an increased risk of CAD and obstructive CAD, after we adjusted for the traditional CV risk factors. However, no IR parameters were related to the increased risk of both CAD and obstructive CAD in diabetic patients. The HbA1C level was an independent risk factor of CAD and obstructive CAD in these subjects. This is consistent with the results of a recent long-term follow-up study emphasizing the significance of strict glucose control for reducing CV events in patients with established diabetes^7–10^. This finding suggests that both IR and concomitant atherogenic dyslipidemia significantly influence subclinical coronary atherosclerosis in non-diabetic people^32,33^, but the main mechanism of the development and progression of coronary atherosclerosis may be strongly linked to chronic exposure to hyperglycemia in patients with established diabetes^34,35^.

Previous several studies evaluated the relationship of IR and glycemic status to CAD in symptomatic patients who referred to coronary angiography^5,6^. However, there is a paucity of data on this issue in asymptomatic individuals. In clinical practice, it is hard to perform coronary angiography for evaluating coronary atherosclerosis in asymptomatic population because of its expensiveness and invasiveness. Recently, CCTA has been established as a novel non-invasive imaging tool that demonstrates high diagnostic accuracy for the detection of CAD and has an effective prognostic utility to predict major adverse cardiac events^36–39^. Compared with previous studies, the present study has several strengths in that (1) various IR parameters were used and (2) the impact of IR parameters and glycemic status on coronary atherosclerosis according to the presence of diabetes was evaluated in asymptomatic large population using CCTA.

The present study has some limitations. First, it had a retrospective design and was based on healthy people who underwent a general health check-up examination. Thus, the results might be influenced by selection biases or unobserved confounders. Second, we could not eliminate the possible effects of medications on coronary atherosclerosis because of the observational design of the study. Third, our participants were exclusively Korean population. Therefore, it might be hard to generalize our findings to other ethnic groups. Finally, we did not investigate the relationship between IR parameters and the adverse characteristics of plaque, such as positive remodeling, low plaque attenuation and spotty calcification^40^. Despite the limitations of the present study, it is unique because we identified the relationship of IR parameters and glycemia with the presence and severity of CAD according to diabetes in a large sample of asymptomatic patients.

In conclusion, our data suggest that IR parameters, especially TyG index, is independently associated the CAD and obstructive CAD in non-diabetics. However, regarding the presence and severity of CAD, glycemic status is more related than the IR in established diabetics. These results could help clinicians understand the different association among IR, glycemia, and coronary atherosclerosis according to the presence of diabetes.

## Supplementary information

Supplementary table 1 & 2

## Data Availability

The datasets used and analyzed during the current study are available from the corresponding author on reasonable request.

## References

[1] Smith SC (2004). Principles for national and regional guidelines on cardiovascular disease prevention: a scientific statement from the World Heart and Stroke Forum. Circulation.

[2] An X (2012). Insulin resistance predicts progression of de novo atherosclerotic plaques in patients with coronary heart disease: a one year follow-up study. Cardiovasc. Diabetol..

[3] Iguchi T (2014). Insulin resistance is associated with coronary plaque vulnerability: insight from optical coherence tomography analysis. Eur. Heart J. Cardiovasc. Imaging.

[4] Uetani T (2012). Impact of insulin resistance on post-procedural myocardial injury and clinical outcomes in patients who underwent elective coronary interventions with drug-eluting stents. JACC. Cardiovasc. Interv..

[5] Mossmann M (2015). HOMA-IR is associated with significant angiographic coronary artery disease in non-diabetic, non-obese individuals: a cross-sectional study. Diabetol. Metab. Syndr..

[6] Verdoia M (2014). Glycosylated hemoglobin and coronary artery disease in patients without diabetes mellitus. Am. J. Prev. Med..

[7] Holman RR, Paul SK, Bethel MA, Matthews DR, Neil HA (2008). 10-year follow-up of intensive glucose control in type 2 diabetes. N. Engl. J. Med..

[8] Hayward RA (2015). Follow-up of glycemic control and cardiovascular outcomes in type 2 diabetes. N. Engl. J. Med..

[9] Orchard TJ (2015). Association between 7 years of intensive treatment of type 1 diabetes and long-term mortality. JAMA..

[10] Zhao W (2014). HbA1c and coronary heart disease risk among diabetic patients. Diabetes Care.

[11] Guerrero-Romero F (2010). The product of triglycerides and glucose, a simple measure of insulin sensitivity. Comparison with the euglycemic-hyperinsulinemic clamp. J. Clin. Endocrinol. Metab..

[12] McLaughlin T (2005). Is there a simple way to identify insulin-resistant individuals at increased risk of cardiovascular disease?. Am. J. Cardiol..

[13] Vega GL, Barlow CE, Grundy SM, Leonard D, DeFina LF (2014). Triglyceride-to-high-density-lipoprotein-cholesterol ratio is an index of heart disease mortality and of incidence of type 2 diabetes mellitus in men. J. Investig. Med..

[14] American Diabetes Association. Standards of medical care in diabetes—2014. *Diabetes Care* **37**(Suppl. 1), S14–S80 (2014). 10.2337/dc14-S014 24357209

[15] Furusyo N (2011). Utility of glycated albumin for the diagnosis of diabetes mellitus in a Japanese population study: results from the Kyushu and Okinawa Population Study (KOPS). Diabetologia.

[16] Matthews DR (1985). Homeostasis model assessment: insulin resistance and beta-cell function from fasting plasma glucose and insulin concentrations in man. Diabetologia.

[17] Simental-Mendia LE, Rodriguez-Moran M, Guerrero-Romero F (2008). The product of fasting glucose and triglycerides as surrogate for identifying insulin resistance in apparently healthy subjects. Metab. Syndr. Relat. Disord..

[18] Eddy D, Schlessinger L, Kahn R, Peskin B, Schiebinger R (2009). Relationship of insulin resistance and related metabolic variables to coronary artery disease: a mathematical analysis. Diabetes Care.

[19] Hanley AJ, Williams K, Stern MP, Haffner SM (2002). Homeostasis model assessment of insulin resistance in relation to the incidence of cardiovascular disease: the San Antonio Heart Study. Diabetes Care.

[20] Bonora E (2007). Insulin resistance as estimated by homeostasis model assessment predicts incident symptomatic cardiovascular disease in caucasian subjects from the general population: the Bruneck study. Diabetes Care.

[21] Hedblad B, Nilsson P, Engstrom G, Berglund G, Janzon L (2002). Insulin resistance in nondiabetic subjects is associated with increased incidence of myocardial infarction and death. Diabet. Med..

[22] Robins SJ (2003). Insulin resistance and cardiovascular events with low HDL cholesterol: the Veterans Affairs HDL Intervention Trial (VA-HIT). Diabetes Care.

[23] Yanase M (2004). Insulin resistance and fasting hyperinsulinemia are risk factors for new cardiovascular events in patients with prior coronary artery disease and normal glucose tolerance. Circ. J..

[24] Tenenbaum A (2007). Insulin resistance is associated with increased risk of major cardiovascular events in patients with preexisting coronary artery disease. Am. Heart J..

[25] Bonora E (2002). HOMA-estimated insulin resistance is an independent predictor of cardiovascular disease in type 2 diabetic subjects: prospective data from the Verona Diabetes Complications Study. Diabetes Care.

[26] Adler AI (2005). Insulin sensitivity at diagnosis of type 2 diabetes is not associated with subsequent cardiovascular disease (UKPDS 67). Diabet. Med..

[27] Wallace TM, Matthews DR (2002). The assessment of insulin resistance in man. Diabet. Med..

[28] Guerrero-Romero F (2016). Fasting triglycerides and glucose index as a diagnostic test for insulin resistance in young adults. Arch. Med. Res..

[29] Er LK (2016). Triglyceride glucose-body mass index is a simple and clinically useful surrogate marker for insulin resistance in nondiabetic individuals. PLoS One.

[30] Vasques AC (2011). TyG index performs better than HOMA in a Brazilian population: a hyperglycemic clamp validated study. Diabetes Res. Clin. Pract..

[31] Lee SH (2014). Predicting the development of diabetes using the product of triglycerides and glucose: the Chungju metabolic disease cohort (CMC) study. PLoS One.

[32] Lim S (2011). Effect of metabolic syndrome on coronary artery stenosis and plaque characteristics as assessed with 64-detector row cardiac CT. Radiology.

[33] Park GM (2015). Impact of metabolic syndrome on subclinical atherosclerosis in asymptomatic individuals. Circ. J..

[34] Meigs JB (2002). Coronary artery calcification in type 2 diabetes and insulin resistance: the framingham offspring study. Diabetes Care.

[35] Anand DV (2007). Determinants of progression of coronary artery calcification in type 2 diabetes role of glycemic control and inflammatory/vascular calcification markers. J. Am. Coll. Cardiol..

[36] Meijboom WB (2008). Diagnostic accuracy of 64-slice computed tomography coronary angiography: a prospective, multicenter, multivendor study. J. Am. Coll. Cardiol..

[37] Budoff MJ (2008). Diagnostic performance of 64-multidetector row coronary computed tomographic angiography for evaluation of coronary artery stenosis in individuals without known coronary artery disease: results from the prospective multicenter ACCURACY (Assessment by Coronary Computed Tomographic Angiography of Individuals Undergoing Invasive Coronary Angiography) trial. J. Am. Coll. Cardiol..

[38] Min JK (2011). Age- and sex related differences in all-cause mortality risk based on coronary computed tomography angiography findings results from the International Multicenter CONFIRM (Coronary CT Angiography Evaluation for Clinical Outcomes: An International Multicenter Registry) of 23,854 patients without known coronary artery disease. J. Am. Coll. Cardiol..

[39] Chow BJ (2011). Incremental prognostic value of cardiac computed tomography in coronary artery disease using CONFIRM: COroNary Computed Tomography Angiography Evaluation for Clinical Outcomes: an InteRnational Multicenter registry. Circ. Cardiovasc. Imaging.

[40] Motoyama S (2009). Computed tomographic angiography characteristics of atherosclerotic plaques subsequently resulting in acute coronary syndrome. J. Am. Coll. Cardiol..

