# Supplementary material for: Association between insulin resistance, hyperglycemia, and coronary artery disease according to the presence of diabetes
Source: Sci Rep. 2019 Sep 2;9:6129. doi: 10.1038/s41598-019-42700-1 (PMC6718672; doi:10.1038/s41598-019-42700-1)
Supplement: Supplementary file 1 — Supplementary table 1 & 2 [file 41598_2019_42700_MOESM1_ESM.pdf]

Association between insulin resistance, hyperglycemia, and coronary artery disease according to the presence of diabetes

Young-Rak Cho[1], Soe Hee Ann[2], Ki-Bum Won[2], Gyung-Min Park[2], Yong-Giun Kim[2], Dong Hyun Yang[3], Joon-Won Kang[3], Tae-Hwan Lim[3], Hong-Kyu Kim[4], Jaewon Choe[4], Seung-Whan Lee[5], Young-Hak Kim[5] , Shin-Jae Kim[2] and Sang-Gon Lee[2]

[1]Division of Cardiology, Dong-A University Hospital, Busan, Republic of Korea

[2]Division of Cardiology, Ulsan University Hospital, University of Ulsan College of Medicine, Ulsan, Republic of Korea

[3]Division of Radiology, Asan Medical Center, University of Ulsan College of Medicine, Seoul, Republic of Korea

[4]Division of Health Screening and Promotion Center, Asan Medical Center, University of Ulsan College of Medicine, Seoul, Republic of Korea

[5]Division of Cardiology, Asan Medical Center, University of Ulsan College of Medicine, Seoul, Republic of Korea

Young-Rak Cho[1], E-mail: nephrone@hanmail.net

Soe Hee Ann[2], E-mail: ash@uuh.ulsan.kr

Ki-Bum Won[2*], E-mail: kbwon99@gmail.com

Gyung-Min Park[2], E-mail: min8684@hanmail.net

Yong-Giun Kim[2], E-mail: apollo0822@naver.com

Dong Hyun Yang[3], E-mail: donghyun.yang@gmail.com

Joon-Won Kang[3], E-mail: jwonkang@amc.seoul.kr

Tae-Hwan Lim[3], E-mail: thlim@amc.seoul.kr

Hong-Kyu Kim[4], E-mail: hkkim0801@amc.seoul.kr

Jaewon Choe[4], E-mail: drchoe@hotmail.com

Seung-Whan Lee[5], E-mail: seungwlee@amc.seoul.kr

Young-Hak Kim[5], E-mail: mdyhkim@amc.seoul.kr

Shin-Jae Kim[2], E-mail: kimsc226@uuh.ulsan.kr

Sang-Gon Lee2, E-mail: sglee@uuh.ulsan.kr

Author contributorship

The first two authors contributed equally to the manuscript’s preparation.

Running title: IR, hyperglycemia, and CAD according to diabetes

Data previously presented: None

Total word count: 2,530

Number of tables and figures: 4 tables and 1 figure

Correspondence to:

Ki-Bum Won, MD

Division of Cardiology, Ulsan University Hospital, University of Ulsan College of Medicine

877 Bangeojinsunhwando-ro, Dong-gu, Ulsan, 44033, Republic of Korea

Telephone: +82(0)522508988

Fax: +82(0)522507048

E-mail: kbwon99@naver.com

Supplementary table 1 The cut-off values for quartiles of HOMA-IR and TyG index according to diabetic status

	Non-diabetics		Diabetics	
Quartiles	HOMA-IR	TyG index	HOMA-IR	TyG index
I (lowest)	0.215−1.136	6.978−8.238	0.315−1.629	7.518−8.636
II	1.137−1.668	8.239−8.595	1.630−2.469	8.637−9.041
III	1.669−2.486	8.596−8.974	2.470−3.926	9.042−9.490
IV (highest)	2.487−16.506	8.975−11.033	3.927−80.766	9.491−11.828

HOMA-IR homeostatic model assessment of insulin resistance; *TyG* triglyceride-glucose

Supplementary table 2 Univariate logistic regression analysis for the association between clinical variables and coronary plaques according to diabetic status

	**Non-diabetics**				**Diabetics**			
	CAD		Obstructive CAD		CAD		Obstructive CAD	
	OR (95% CI)	p	OR (95% CI)	p	OR (95% CI)	p	OR (95% CI)	p
Age, years	1.095 (1.085−1.105)	<0.001	1.088 (1.072−1.105)	<0.001	1.082 (1.062−1.102)	<0.001	1.064 (1.040−1.089)	<0.001
Male	3.404 (2.937−3.945)	<0.001	3.215 (2.268−4.557)	<0.001	1.771 (1.276−2.460)	0.001	1.067 (0.671−1.699)	0.783
Waist circumference, cm	1.050 (1.042−1.058)	<0.001	1.036 (1.021−1.050)	<0.001	1.022 (1.006−1.039)	0.007	1.002 (0.981−1.024)	0.838
Hypertension	2.485 (2.195−2.812)	<0.001	2.657 (2.109−3.348)	<0.001	2.067 (1.599−2.672)	<0.001	1.611 (1.126−2.303)	0.009
Dyslipidemia	1.746 (1.538−1.983)	<0.001	1.851 (1.466−2.339)	<0.001	1.414 (1.094−1.827)	0.008	1.268 (0.895−1.798)	0.182
Current smoking	1.177 (1.026−1.351)	0.020	1.093 (0.836−1.428)	0.515	1.189 (0.900−1.570)	0.223	1.022 (0.699−1.493)	0.912
HOMA-IR	1.167 (1.112−1.224)	<0.001	1.145 (1.060−1.237)	0.001	1.093 (1.035−1.154)	0.001	1.037 (1.003−1.072)	0.035
TyG index	1.631 (1.459−1.822)	<0.001	1.686 (1.368−2.078)	<0.001	1.094 (0.900−1.330)	0.366	1.341 (1.032−1.743)	0.028
TG/HDL	1.098 (1.068−1.130)	<0.001	1.104 (1.059−1.151)	<0.001	0.999 (0.955−1.045)	0.979	1.064 (1.008−1.123)	0.025
HbA1C, %	1.682 (1.438−1.968)	<0.001	1.817 (1.334−2.474)	<0.001	1.227 (1.095−1.375)	<0.001	1.395 (1.234−1.577)	<0.001

CAD coronary artery disease, *CI* confidence interval, *HbA1C* hemoglobin A1C, *HDL* high-density lipoprotein, *HOMA-IR* homeostatic model assessment of insulin resistance, *OR* odds ratio, *TG* triglyceride, *TyG* triglyceride-glucose
